# Community and Campus COVID-19 Risk Uncertainty Under University Reopening Scenarios: Model-Based Analysis

**DOI:** 10.2196/24292

**Published:** 2021-04-07

**Authors:** James Benneyan, Christopher Gehrke, Iulian Ilies, Nicole Nehls

**Affiliations:** 1 Healthcare Systems Engineering Institute Northeastern University Boston, MA United States

**Keywords:** COVID-19, university reopening, community impact, epidemic model, model, community, university, safety, strategy, risk, infectious disease

## Abstract

**Background:**

Significant uncertainty has existed about the safety of reopening college and university campuses before the COVID-19 pandemic is better controlled. Moreover, little is known about the effects that on-campus students may have on local higher-risk communities.

**Objective:**

We aimed to estimate the range of potential community and campus COVID-19 exposures, infections, and mortality under various university reopening plans and uncertainties.

**Methods:**

We developed campus-only, community-only, and campus × community epidemic differential equations and agent-based models, with inputs estimated via published and grey literature, expert opinion, and parameter search algorithms. Campus opening plans (spanning fully open, hybrid, and fully virtual approaches) were identified from websites and publications. Additional student and community exposures, infections, and mortality over 16-week semesters were estimated under each scenario, with 10% trimmed medians, standard deviations, and probability intervals computed to omit extreme outliers. Sensitivity analyses were conducted to inform potential effective interventions.

**Results:**

Predicted 16-week campus and additional community exposures, infections, and mortality for the base case with no precautions (or negligible compliance) varied significantly from their medians (4- to 10-fold). Over 5% of on-campus students were infected after a mean of 76 (SD 17) days, with the greatest increase (first inflection point) occurring on average on day 84 (SD 10.2 days) of the semester and with total additional community exposures, infections, and mortality ranging from 1-187, 13-820, and 1-21 per 10,000 residents, respectively. Reopening precautions reduced infections by 24%-26% and mortality by 36%-50% in both populations. Beyond campus and community reproductive numbers, sensitivity analysis indicated no dominant factors that interventions could primarily target to reduce the magnitude and variability in outcomes, suggesting the importance of comprehensive public health measures and surveillance.

**Conclusions:**

Community and campus COVID-19 exposures, infections, and mortality resulting from reopening campuses are highly unpredictable regardless of precautions. Public health implications include the need for effective surveillance and flexible campus operations.

## Introduction

The COVID-19 pandemic has had devastating human, financial, and logistical impacts worldwide, including over 125 million infected and 2.75 million deaths as of March 2021 [1], radical changes to work and life routines, economic recession, and increased social inequities [2-5]. Among many other issues, significant uncertainties exist about the potential safety and consequences of reopening schools [6-9], heightened by resurgences in infections and mortality and campus × community cross-exposure concerns [9,10]. Although much initial focus was on K-12 education [11-13], similar college and university reopening concerns exist [9,10,14].

As COVID-19 spread uncontrollably during the spring of 2020, nearly all K-12 and postsecondary schools suspended physical classes, with an estimated 50 million elementary students [13] and 19 million college students in the United States [15] shifting to online learning, homeschooling, and remote education, with experiences varied and often lacking [16-18]. Although a few universities decided early in summer 2020 to remain fully virtual for the following academic year, including the largest public university system in the United States [19,20], many schools decided to reopen under various structures. Since then, four events of import have occurred: several additional colleges and universities switched to full or partial online operations for the fall 2020 semester; COVID-19 has resurged in many regions; other schools have committed to opening as safely as possible; and debate has increased as to what best balances education, safety, and economic needs [21-26].

Examples of reopening approaches range from full on-campus operations with contact precautions; hybrid virtual/physical formats with some courses (or class meetings within given courses) taught virtually and others in-person; having only first- and/or second-year students on campus with all others virtual; student choice to take courses physically versus virtually; and (in the United States) accelerated semesters ending at the Thanksgiving holiday to reduce travel-based spread [27-29]. Efforts to limit on-campus exposures include reconfigured classrooms and dormitory spaces, precaution awareness campaigns, hotel room rentals to reduce living density, testing and tracing plans of varied rigor, isolation of returning students, dedicated living spaces for students with positive tests, and other strategies that attempt to reduce density and exposure rates [21,23,27,30-32].

Significant uncertainty, however, exists about the effectiveness of any of these plans [21,23,31,33]. The best current diagnostic tests have variable and poor clinical sensitivity [25,34] and turnaround delays, while incubation from the time of exposure to becoming symptomatic averages 3-5 days [35-37]. Furthermore, an estimated 30%-40% of positive individuals never exhibit symptoms [25,34,35], and on-campus compliance to distancing precautions generally is low [25,30,38,39]. Contact tracing, while helpful, may not work as well for COVID-19 given the above [9,33] and may be further limited in the campus context as students interact with many-fold more individuals (many unknowingly or unknown by name).

These uncertainties have prompted some to question university reopening safety [6,8,14,25,26,31,40], especially in urban university settings with significant geographically dispersed student populations [41]. Others have suggested COVID-19 might catalyze the reinvention of higher education [42-45], including criticisms of prioritizing economics, brand, and survival over safety [22,43,44,46]. The president of Paul Quinn College, by example, stated “Rushing to reopen our society and our schools is a mistake that will ultimately result in hundreds of thousands of citizens falling sick and worse. We should not let our own financial and reputational worries cloud our judgment about matters of life and death” [8]. In contrast, not reopening may have large economic and student development effects [47-49], although perhaps less of both effects compared to not reopening K-12 schools. Not reopening could also be untenable for colleges and universities that were already facing financial strains before COVID-19 emerged [48-50].

Although little empirical data exist on college reopening [40,51-56], experiences of preschool, summer camp, and K-12 programs have been varied [57,58], with some outbreaks traced back to only a few index cases [21]. Social gatherings of college-age students during summer 2020 also have resulted in outbreaks [38,58,59], including events and activities individuals were advised against but participated in nonetheless [30,38]. Despite early uncertainty, increasing evidence suggests student-aged individuals can carry and transmit the SARS-CoV-2 virus [35,58,60,61] and significant between-student spread occurs at college and high school levels [35,58,61] (in contrast to younger K-5 students [35,47,61,62]). The impact of campus opening on spread to the surrounding community, with higher percentages of at-risk individuals, has been less reported on.

Given these combined uncertainties, we developed single and multiple population COVID-19 spread models to investigate the predictability of potential community and campus impacts under various reopening scenarios. The intent is to provide model-based analysis to better inform decision-making at a critical time in the COVID-19 pandemic. Although similar model analyses have extensively studied other infectious disease policies [63-66], there has been little investigation of university reopening and the impact on surrounding communities.

## Methods

### Model Overview

We developed and validated single and multiple population ordinary differential equations (ODE) and agent-based models of COVID-19 spread within and between defined groups of individuals. The general model logic (Multimedia Appendix 1) was adapted from classic susceptible-exposed-infected-recovered (SEIR) frameworks [67,68] similar to those described elsewhere for many other infectious diseases [63-76]. The single population model describes spread dynamics within one defined population (eg, on-campus students or local community residents), whereas the multipopulation model additionally includes cross-exposure between two or more groups. Multiple change points were included for all parameters to allow for policy or behavior changes when fitting models to historical data.

State variables at time *t* include the numbers of individuals in population *j* that are free of and susceptible to COVID-19 (*S_j_*(*t*)), exposed to COVID-19 but not yet infectious themselves (*E_j_*(*t*)), COVID-19–positive and infectious to others (*I_j_*(*t*)), recovered and not susceptible to reinfection (*R_j_*(*t*)), and COVID-19–associated deceased (*D_j_*(*t*)). Exposed individuals are assumed to have a small chance (*p*) of warding off an infection before becoming infectious. Recovered individuals are assumed not able to be reinfected within at least a 16-week (one semester) time frame [37,77]. Each state variable is updated numerically at each time increment (set here to 0.01 days) based on its previous value, values of other state variables at the previous time step, and the equations governing their interdependent relationships, with this process continuing iteratively for 16 weeks.

For example, the number of individuals in the susceptible population (*S_j_*(*t*)) is decremented by the number of newly exposed individuals (*S_j_*(*t*) · *expo_j_*) and increased by the number who previously were exposed but did not develop infections, (*E_j_*(*t*) · *expr_j_*), where the daily exposure rate *expo_j_*, the average risk of transmission multiplied by the average number of contacts per day, is back-computed from the basic reproduction number *R_0_* (average number of new infections per infected individual) and recovery and mortality rates, and the recovery rate of noninfectious exposed individuals *expr_j_* is the inverse of the corresponding recovery time *t_rec, nonj_*.

In turn, the number of exposed individuals is increased by *S_j_*(*t*) · *expo_j_* and decremented by the number who develop infections (*E_j_*(*t*) · *infe_j_*), where the daily infection rate *infe_j_* is the ratio of the probability of becoming infected upon exposure *p_j_*over the average incubation time *t_incj_*. Infected individuals either recover or die at rates of *infr_j_* · *I_j_*(*t*) and *mort_j_* · *I_j_*(*t*), respectively, where the daily recovery and mortality rates are the inverse of the average recovery time *t_rec, incj_* and the ratio of the overall COVID-19 case fatality rate for that population (*CFR_j_*) over the average time from infection until death *t_i2dj_*, respectively.

The governing rate change dynamics for each state variable at each time step during numeric evaluation thus are the following:

*S_j_*(*t*) (susceptible; + not-infected/infectious (nor immune) after exposure – new exposures due to within-population and between-population contact with infectious individuals): 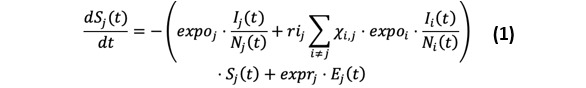


*E_j_*(*t*) (exposed; + new exposures – past exposures now infected/infectious – past exposures now not infected/infectious [now susceptible]): 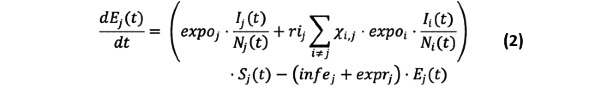


*I_j_*(*t*) (infectious; + past exposures now infected/infectious – past exposures now not infected – deaths): 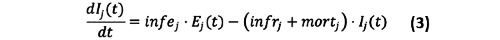


*R_j_*(*t*) (recovered; + infected individuals who recover [with immunity]): 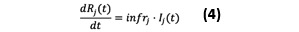


*D_j_*(*t*) (deceased; + COVID-19–related deaths): 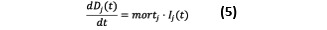


where


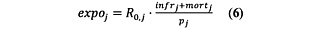
 (rate at which people transition from susceptible to exposed)


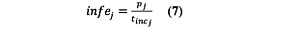
 (rate at which people transition from exposed to infected)


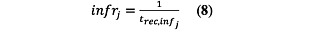
 (rate at which people transition from infected to recovered)


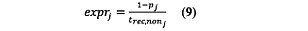
 (rate at which people transition from exposed to recovered)


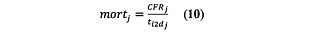
 (rate at which people transition from infected to deceased) and


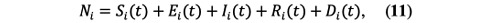
 (sum of all subpopulations in region *i* at time *j*)

where χ*i,j* = 1 if populations *i* and *j* interact and 0 otherwise and *p_j_* = the proportion of exposed individuals that transition to infected (versus recovering to susceptible). The multipopulation models allow for separate parameter values for each population, such as based on their demographics, with a cross-exposure parameter (*ri_j_*) defining the relative rate at which infectious individuals in one population expose susceptible individuals in the other (typically lower than within-population, assuming less interaction).

### Parameter Estimation and Model Calibration

Model accuracy was validated using standard methods [65,78-82], cross-validation, and varied state and county empirical data (January to July 2020) exhibiting different epidemic patterns, magnitudes, and timings (Multimedia Appendix 2). Model results closely emulate historical data across multiple settings, with accuracy on par with or exceeding norms reported elsewhere [83-89] and with ≤1 change points generally providing good fits, suggesting good prospective short-term prediction capability.

Model inputs (Table 1) used in the community and campus models were estimated using a combination of published and grey literature, expert opinion, and search-based optimization. For campus inputs with uncertainty, we used Monte Carlo simulations to create 1000 synthetic results across plausible ranges, using the shown most likely, maximum, and minimum values to generate asymmetric triangular distribution random variates. Since little data exist about on-campus spread [21], for exposure rates we used the shown ranges for the average number of infected students divided by the exposed-to-infectious percentage.

For community populations, we further calibrated inputs via a particle swarm search algorithm to minimize root mean square error differences between historical and model-predicted infections and mortality, running each parameter search 1000 times. For model fits with change points, separate values for all inputs were optimized for each time segment, with state variables at the start of each new time segment set to their values at the end of the prior segment.

**Table 1:** Model parameters in the COVID-19 campus × community epidemic models, estimated values, literature sources, and ranges used for parameter search and sensitivity analysis. “Rank order” indicates the relative significance of each parameter on campus (community; additional community) outcomes (16-week totals); only statistically significant factors are shown (α=.05).

Parameter	Definition	Lower bound	Most likely	Upper bound	Sources	Rank	
						Infection	Mortality
*R_0,1_*	Average number of students who become infected by infectious students	0.66	1	3.4	[90]	2 (6; 3)	5 (—^a^; 3)
*R_0,2_*	Average number of residents infected by infectious residents	0.66	—	3.4	[90], parameter search	5 (2; 2)	— (5; 4)
*ri_j_*	Cross-exposure parameter (campus × community)	0.005	0.008	0.02	Estimated	6 (6; 5)	— (—; 8)
*π_1_*	Proportion of student population initially infected at semester start	0.001	.01	.05	[91-93]	4 (—; 6)	6 (—; 9)
*π_2_*	Proportion of community population initially infected at semester start	0.0016	.01	.016	[94]	— (4; 4)	— (7; 10)
*p_j_*	Proportion of exposed people that become infected	0.5	0.9	1	Estimated	— (7; —)	3 (3; 5)
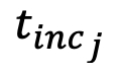	Incubation duration (in days)	2	4.5	14	[95,96]	3 (3; 7)	7 (6; 7)
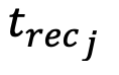	Recovery duration (in days)	6	14	42	[97-99]	1 (1; 1)	2 (1; 1)
*CFR_1_*	Fatality rate for college population	0.001	0.0092	0.016	[97,100]	— (—; —)	1 (—; —)
*CFR_2_*	Fatality rate for community population	0.01	0.06	0.15	[101,102]	— (5; —)	— (2; 2)
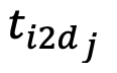	Number of days from infection until death	14	35	56	[99]	— (8; —)	4 (4; 6)

^a^Not available.

For initial disease prevalence in the local community and among arriving students, we also used expected values and probability intervals from a logistic growth curve fit to historical COVID-19 infection counts, estimating prevalence among arriving students at the start of the fall 2020 semester using a weighted average of prevalence predictions based on home locations. Resulting community and student prevalence ranges were validated against data reported in the media. Positive individuals at the start of the semester were assumed distributed between exposed but not yet infectious (24.3%) and infectious (75.7%) groups based on approximate relative durations that an average infected individual might spend in each state. All model inputs were based on published literature listed in Table 1 or aggregate state and county infection and mortality online data [103] and thus not subject to human subjects internal review board approval.

### Reopening Scenario Analysis

Common university reopening scenarios were identified from literature and published surveys [7,19], generally belonging to one of several categories (see Table 2 for examples), which then were used to estimate plausible ranges for *R_0_* reductions. The most common approaches included primarily or fully in-person (35%), primarily or fully online (32%), and hybrid (19%) [7]. As examples, the University of Washington reopening plan [104] exemplifies a conservative approach, with more than 90% of courses taught online, courses relying on direct interactions (eg, medical and health sciences) taught in person with safety precautions, the majority of student services and advising taking place remotely, and any staff who can work remotely doing so. In contrast, Purdue University illustrates an opposite approach [105], with classes mainly taught on campus with contact precautions until the Thanksgiving break, relying on students to manage their personal safety.

**Table 2:** Representative examples of US university and college COVID-19 fall 2020 semester campus reopening plans.

Intervention description	Examples	Source	Estimated reduction in *R*_0_ (%)
Remote coursework for classes over 50, testing, contact tracing, health surveillance in dorms	University of Washington	[104,106]	36
Remote option available, social distancing, shortened semester, flexible start dates for international students	Rice University	[107]	25
Face masks, social distancing, limited classes, some coursework online, fewer students living on campus, shorter semester	Stanford University	[108]	33
More online classes, masks, social distancing, testing, health surveillance, sanitizing and washing stations	Ohio State University	[109,110]	30
Some classes online, expanded housing, social distancing, face masks, staggered hours, increased cleaning, testing, tracing	Northeastern University	[32,111]	49
Most classes online except those for which in-person instruction is deemed necessary	California State University	[112,113]	12
Students back on campus for a shortened fall semester as long as they follow Connecticut reopening suggestions	Connecticut State institutions	[114,115]	46

As most reopening plans involve reducing either interpersonal contact or infection spread, we implemented these as multiplicative reductions in the reproduction number *R_0_*, with effect sizes of individual actions estimated via literature estimates and expert opinion. Overall scenarios then were defined with estimated effects on *R_0_* spanning the base case of no change, small 25% cumulative reductions (eg, Rice University, Ohio State), moderate 50% reductions (eg, Northeastern University, Connecticut State), and 75% reductions as a best-case scenario for comparison, with each scenario coupled with initial student infection rates of 0.1%, 1%, 2%, and 5% based on university reporting.

For each scenario, 1000 model replications were run for campus alone, community alone, and campus × community combined to estimate additional cross-exposure impacts of each population on the other. For the campus × community cases, each of the 1000 community parameterizations were randomly coupled with the 1000 random sets of campus inputs, with the two populations interacting via 1000 random values of the cross-exposure parameter, *ri*, sampled from the range shown in Table 1.

Given that the ratio of campus-to-community population sizes may affect cross-infection results and prevention policies, we assumed three general settings: (1) an urban campus of 10,000 students with 100,000 residents living in the immediately surrounding residential areas or neighborhoods (in which off-campus students tend to reside); (2) a student body of the same size (10,000), but with fewer (40,000) residents living close to the campus; and (3) a smaller number of 2000 students with 40,000 residents living near campus. The first scenario might represent a large university in a major city, whereas the second might represent a large rural university, and the third a smaller undergraduate college in a nonurban setting, although these student-to-community populations (1:10, 1:4, and 1:20) can be extrapolated to other settings with similar ratios.

Monthly and total counts of COVID-19 exposures, infections, and deaths for each population were tabulated and plotted longitudinally. First inflection points (dates of steepest increases) for each outcome, scenario, and population were identified numerically, since in diffusion theory interventions after these points tend to be less effective. To estimate cross-exposure effects, pairwise differences were computed between each of the 1000 campus results and their campus × community counterparts, and similarly between each of the 1000 community results and their campus × community counterparts. For all model results, medians, standard deviations, and 95% probability intervals were computed, with 10% trimming to reduce any extreme outlier replicate effects.

Sensitivity analyses were conducted to identify model inputs to which the mean and variance of results are most sensitive via central composite factorial experimental designs [116] as this could inform policy-making, interventions, and target setting. Results were analyzed using general linear models including linear and pairwise interaction terms for each outcome (replication means and variances of total and additional campus and community infections and deaths), with resulting effect coefficients normalized to their corresponding ranges and ranked according to statistical significance.

## Results

### On-Campus/Student Impact

**Figure 1:**
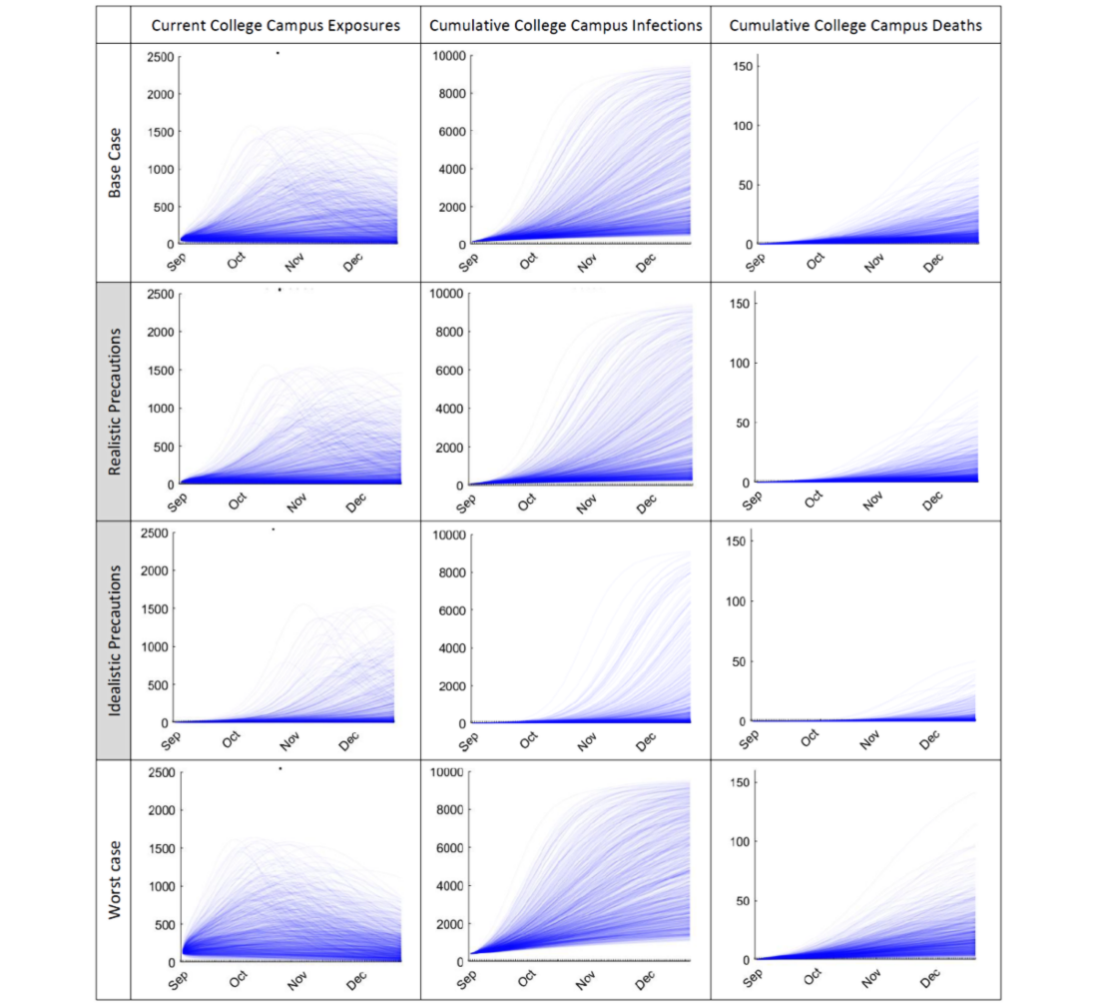
Predicted number of college students per 10,000 who are currently exposed to COVID-19, have been infected to date, and have died to date over a 16-week fall 2020 semester (urban university example). Top row: base case scenario assuming no semester initiation precautions and disease prevalence of 2% among arriving students (equal to national and regional averages). Shaded middle rows (most likely cases): realistic (1%) and idealistic (0.1%) initial prevalence scenarios assuming good or great screening-on-arrival precautions, adherence, and effectiveness. Bottom row: worst case scenario (5% prevalence) assuming little-to-no arrival precautions, compliance, and effectiveness.

Within any given assumptions for COVID-19 prevalence among arriving students and semester initialization precautions, the predicted number of students per 10,000 who might be exposed, be infectious, and die over a 16-week semester could vary by up to 10-fold (Figure 1). By semester end, under the base case (2% arrival prevalence, little returning precautions and/or effectiveness) predicted student outcomes range from 471-9458 infections (median 2286, SD 2627) and 0-123 deaths (median 9, SD 14).

The more realistic case (1% arrival prevalence) reduces these consequences to a median of 1332 (SD 2552) infections and 5 (SD 12) deaths, with the steepest increases in exposures and infections typically occurring at midsemester onward (with important implications on geographic spread as students return to their home communities). Although less likely, idealistic (0.1%) and worst-case (5%) initial prevalence scenarios were also considered for comparison, given that epidemic prevalence might change in future semesters. The first would result in a median of 158 (SD 1760) infections and 1 death (SD 6 deaths) by semester end and the latter in 3996 (SD 2485) infections and 16 (SD 17) deaths.

Note that the left-hand plots in Figure 1 depict the current number of exposed people on any given day (with each exposure spanning several days) in order to give an indication of the changing amount of contract tracing and isolation required, as well as the changing population risk, whereas the other plots depict the cumulative number of infections and deaths to date in order to summarize the total public health impact. Under the base case scenario, the number of active student exposures at any given time (eg, for contact tracing and isolation) ranged from 3-1576 per 10,000 individuals (eg, as high as 15% of a student population), with significant implications on resource planning and viability (Table 3). Under the two most likely scenarios, by midsemester the total number of infections might be as high (mean plus one standard deviation) as 810 per 10,000 students (with 4 deaths) or as low (mean minus one standard deviation) as 782 per 10,000 students (with 0 deaths).

**Table 3:** Predicted median number of monthly COVID-19 exposures, infections, and deaths per 10,000 students during fall 2020 (northeast US urban university example). Values in parentheses indicate 95% probability ranges.

Scenario	September				October				November				December		
	Exposure	Infection	Mortality	Exposure		Infection	Mortality	Exposure		Infection	Mortality	Exposure		Infection	Mortality
Base case	474 (175-1840)	318 (146-1181)	1 (0-3)	708 (160-4731)		510 (131-3561)	2 (1-11)	925 (124-3532)		685 (105-2946)	3 (0-16)	638 (74-1934)		522 (63-1576)	2 (0-12)
Realistic arrival precautions	245 (89-1011)	163 (74-639)	1 (0-2)	392 (84-3723)		278 (68-2777)	1 (0-8)	554 (67-4024)		411 (56-3304)	2 (0-14)	504 (41-2279)		389 (36-1874)	2 (0-12)
Idealistic arrival precautions	26 (9-113)	17 (7-69)	0 (0-0)	44 (9-769)		30 (7-476)	0 (0-1)	70 (7-3072)		49 (6-2133)	0 (0-5)	76 (5-2997)		56 (4-2366)	0 (0-8)
Worst case	1084 (415-3613)	744 (353-2398)	3 (1-8)	1377 (354-4623)		1026 (301-3895)	5 (1-17)	1225 (248-2840)		994 (221-2380)	5 (1-19)	624 (122-1406)		537 (114-1214)	3 (1-12)

**Figure 2:**
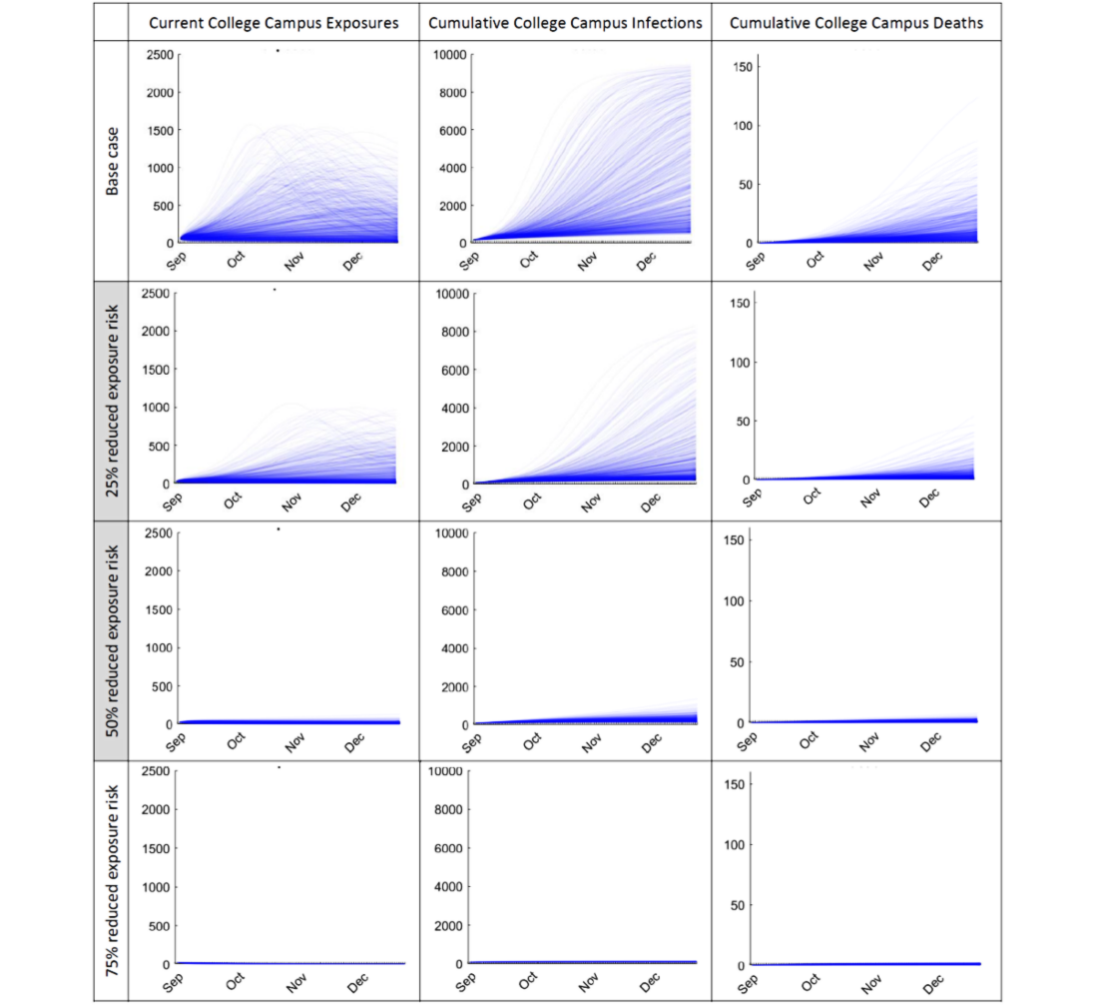
Relative effectiveness of reopening and precaution strategies on reducing college campus student COVID-19 exposures at any given date, total infections to date, and total mortality to date per 10,000 students (fall 2020 semester, assuming 1% of students are infected or exposed at the start of the semester, urban university example). Top row: base case from Figure 1 for comparison; shaded middle rows (most likely cases): realistic precaution effectiveness and compliance cases; bottom row: idealistic precaution effectiveness and compliance. Reduced exposure risk refers to reducing R0.

Similar results occur assuming the various semester precautions summarized in Table 2, corresponding to plausible reductions in *R_0_* of 25%-50% (Figure 2). In general, current strategies to reduce exposure during a semester appear effective, although under most scenarios a concerning number of students still can become infected or die. Even in the very optimistic case of a 75% *R_0_* reduction, included for comparison as a hypothetical “best case” scenario, 95-132 infections (median 107) and 1 death per 10,000 students may occur by midsemester, increasing to 97-139 infections and 0-3 deaths by semester end.

### Community Resident Impact

**Figure 3:**
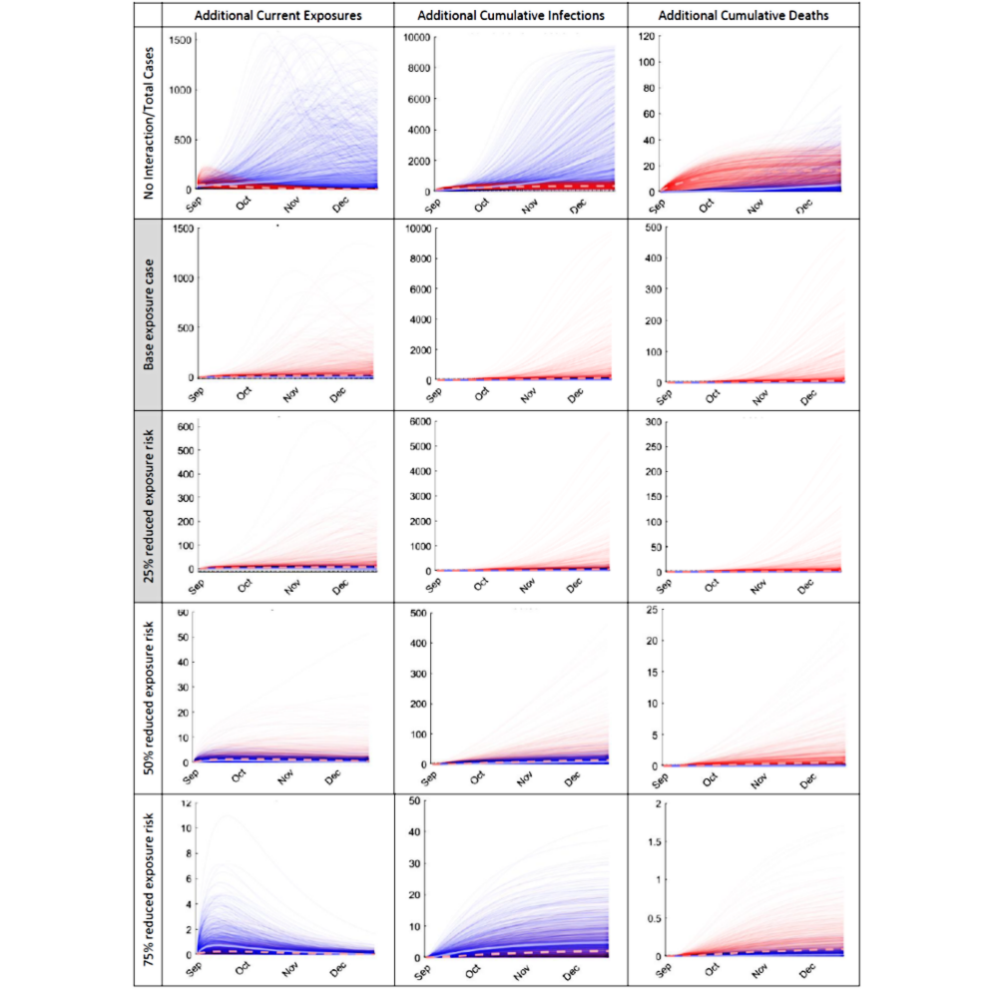
Additional (red) community and (blue) college campus COVID-19 exposures, infections, and mortality due to community × campus cross-exposure (fall 2020 semester, prevalence among arriving students varied between 0.1%-2%, urban university example). No interaction: total outcomes assuming no interaction between school and community. Base case: additional outcomes due to campus reopening assuming little-to-no campus semester operation precautions, compliance, or effectiveness. Shaded rows (most likely cases): additional outcomes assuming likely and ideal cases for campus operation precautions, adherence, and effectiveness. Bottom row: additional outcomes under a best case scenario assuming very high campus semester operation precautions, compliance, and effectiveness.

Figure 3 summarizes additional community (blue lines) and campus (red lines) impacts of reopening due to campus × community cross-exposure, assuming the same scenarios described above; for comparison, the top row shows the baseline number of community exposures, infections, and mortality without reopening. Local community impacts (Table 4) of opening with little-to-no semester operation precautions and/or adherence might range from 1-9768 additional community infections (median 158, SD 1131) and 0-491 additional community deaths (median 6, SD 53).

The two more realistic scenarios result in a total of (for 25% exposure reduction) 1-5577 additional community infections (median 56, SD 516) and 0-272 additional community deaths (median 3, SD 24), and (for 50% exposure reduction) 0-464 additional community infections (median 14, SD 45) and 0-23 additional community deaths (median 1, SD 2). For comparison, the hypothetical best-case scenario with 75% exposure reduction results in 0-33 additional community infections (median 2, SD 4) and 0-2 additional community deaths (median 0, SD 0.2).

**Table 4:** Predicted monthly total campus and community COVID-19 outcomes (per 10,000) during the fall 2020 semester. (A) Total community resident outcomes assuming no university interaction, (B) additional community resident outcomes due to campus × community spread, and (C) additional university student outcomes (urban university example). Tabulated values are medians across 1000 replicates; values in parentheses indicate 95% probability ranges.

Scenario (exposure risk)		September				October				November				December		
		Exposure	Infection	Mortality	Exposure		Infection	Mortality	Exposure		Infection	Mortality	Exposure		Infection	Mortality
**(A) Community outcomes assuming no university interaction (per 10,000)**																
	Monthly totals	11 (0-64)	158 (122-362)	6 (4-18)	4 (0-30)		175 (122-455)	7 (4-26)	1 (0-14)		181 (122-486)	7 (4-28)	1 (0-8)		182 (122-495)	7 (4-29)
**(B) Additional community outcomes due to campus reopening (total)**																
	Base case	6 (1-24)	9 (2-26)	0 (0-1)	11 (1-69)		32 (6-126)	1 (0-4)	13 (1-148)		78 (10-415)	3 (0-10)	14 (1-180)		124 (14-755)	5 (1-19)
	25% reduced exposure	3 (1-14)	6 (2-17)	0 (0-1)	4 (0-23)		17 (4-56)	1 (0-2)	6 (0-42)		32 (5-147)	1 (0-5)	6 (0-57)		48 (7-263)	2 (0-7)
	50% reduced exposure	1 (0-4)	3 (1-7)	0 (0-0)	1 (0-3)		6 (1-16)	0 (0-1)	1 (0-3)		9 (2-23)	0 (0-1)	0 (0-2)		10 (2-29)	1 (0-1)
	75% reduced exposure	0 (0-1)	1 (0-2)	0 (0-0)	0 (0-0)		1 (0-3)	0 (0-0)	0 (0-0)		1 (0-4)	0 (0-0)	0 (0-0)		1 (0-4)	0 (0-0)
**(C) Additional university outcomes due to campus reopening (total)**																
	Base case	0 (0-5)	1 (0-10)	0 (0-0)	1 (0-8)		3 (0-30)	0 (0-0)	0 (0-10)		5 (0-56)	0 (0-0)	0 (0-8)		6 (0-76)	0 (0-0)
	25% reduced exposure	0 (0-4)	1 (0-9)	0 (0-0)	0 (0-5)		2 (0-22)	0 (0-0)	1 (0-5)		3 (0-36)	0 (0-0)	0 (0-5)		4 (0-46)	0 (0-0)
	50% reduced exposure	0 (0-3)	1 (0-7)	0 (0-0)	0 (0-2)		1 (0-14)	0 (0-0)	0 (0-1)		1 (0-19)	0 (0-0)	0 (0-1)		2 (0-21)	0 (0-0)
	75% reduced exposure	0 (0-2)	1 (0-6)	0 (0-0)	0 (0-1)		1 (0-11)	0 (0-0)	0 (0-0)		1 (0-13)	0 (0-0)	0 (0-0)		1 (0-13)	0 (0-0)

The corresponding impact of the community on student outcomes is smaller, ranging from 0-390 additional student infections (median 21, SD 38) and 0-2 additional student deaths (median 0, SD 0.2) for the base case to 0-42 additional student infections (median 5, SD 6) and 0 additional student deaths (median 0, SD 0.04) for the idealistic case (75% exposure reduction). The two more likely cases result in (for 25% exposure reduction) 0-279 additional student infections (median 17, SD 30) and 0-1 additional student deaths (median 0, SD 0.1), and (for 50% exposure reduction) 0-115 additional student infections (median 8, SD 12) and 0-1 additional student deaths (median 0, SD 0.06).

**Figure 4:**
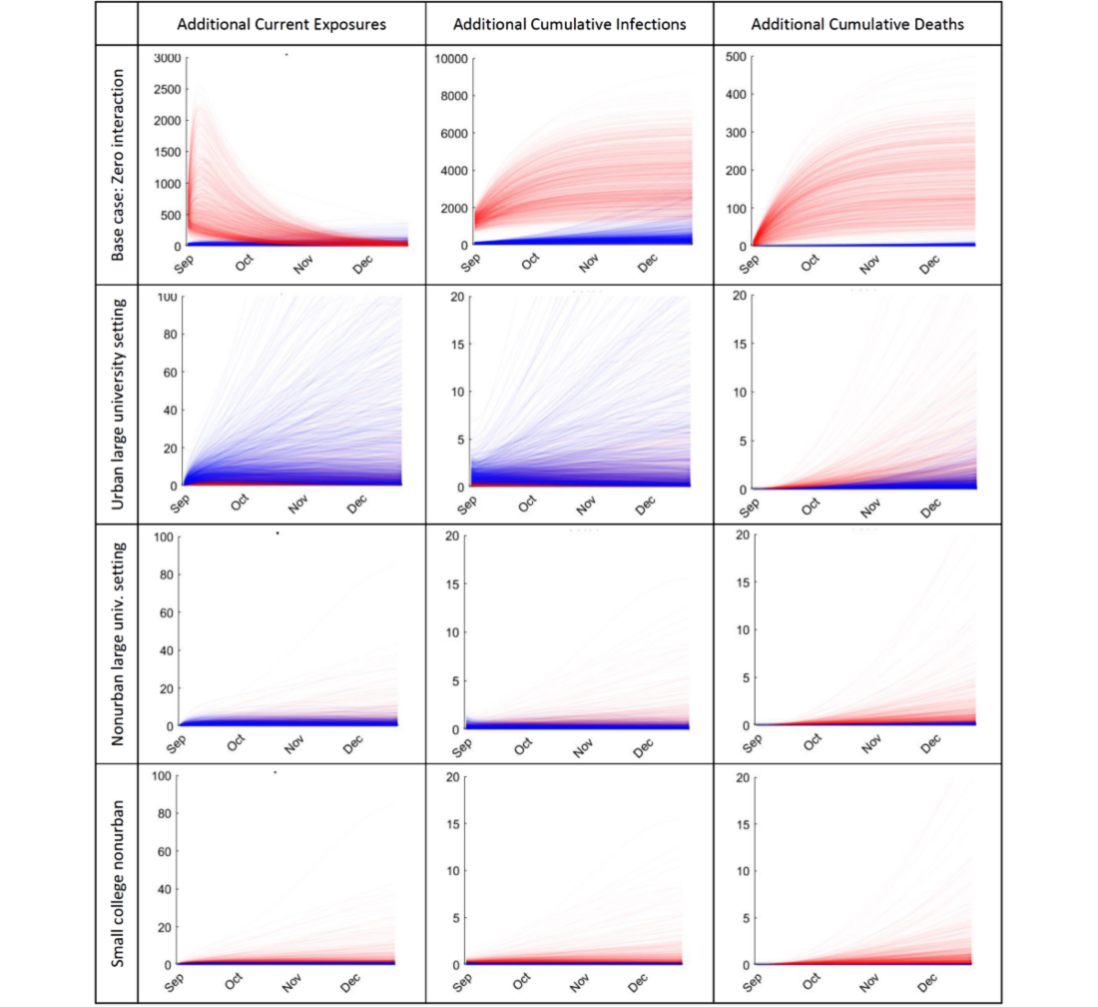
Impact of school-to-community population sizes on predicted additional community resident (red) and student (blue) COVID-19 current exposures, total infections, and total deaths per 10,000 individuals during the fall 2020 semester, assuming 1% prevalence among returning students and effective campus operations precautions (50% R0 reduction). Urban large university: 10,000 students, 100,000 community residents; nonurban large university: 10,000 students, 40,000 community residents; small college nonurban: 2000 students, 40,000 community residents.

To estimate the impact of school size and location, Figure 4 compares results under other student-to-community population sizes, assuming the same arrival prevalence and campus operation precautions, compliance, and effectiveness scenarios. While intuitive differences exist in raw totals, results are similar and scale-invariant after adjusting for population size. For example, multiplying results for the second case of 40,000 residents by 2.5 yields similar curves to those for the first case of 100,000 residents. This suggests that the above results may generalize to other settings and that between-location differences in epidemic patterns (and therefore in public policies to limit spread) likely arise from variations in campus × community interaction, rather than in population ratios.

Finally, factorial sensitivity analysis produced the relative parameter rankings shown in the two rightmost columns of Table 2, which follow intuition and serve as further model validation. The most statistically significant factors (main effects) affecting the expected (Multimedia Appendix 3) and variation (Multimedia Appendix 4) in total campus infections were recovery time, *R_0,campus_*, incubation time, initial prevalence among arriving students, and *R_0,community_*. Similarly, for total community infections, the most important factors were recovery time, *R_0,community_*, incubation time, initial prevalence among community residents, and community case fatality rate*.* Numerous interaction terms were also significant in both cases, as would be expected in such a model.

For additional community infections, no dominant factors that affect either magnitude or variability of outcomes were evident beyond the basic reproduction numbers of each population (*R_0,campus_, R_0,community_*) and the campus × community cross-exposure rate *ri*. The large number of other statistically significant main effects and interaction terms also underscore the multidimensional challenge of predictably limiting community impact and by extension the importance of effective surveillance and mitigation.

## Discussion

### Principal Findings

The COVID-19 pandemic continues to be a significant public health crisis, with infections and mortality in many regions meeting or exceeding those in early 2020 before physical distancing and closures were implemented. With many colleges and universities reopening, model-based analyses can help inform these important decisions as well as the degree of uncertainty in the resulting outcomes. Three important results of the present analysis are the following: (1) infections and mortality from campus reopening are highly variable and nearly impossible to predict with any certainty, (2) reopening campuses can significantly impact local communities even under best-case scenarios, and (3), while few exist, prevention and public health measures that target campus × community exposure could be effective.

While conditions may exist under which reopening is relatively safe to the local community, at present these appear in the significant minority. Our results also agree in general with emerging empirical data from the fall 2020 semester, including reports that COVID-19 deaths in US communities with open colleges roughly doubled from August to December 2020, compared with a smaller 58% average increase in communities without colleges. Genetic sequencing results further suggest that many deaths in college towns were of older people who had contact with infected students [117].

Several important public health implications of our results exist. First, decisions about whether to open in future academic terms or epidemics should be informed by updated model inputs, projections of local conditions, and campus × community public health measures. Second, since any trajectory within the produced intervals could occur, reopening decisions should consider these ranges rather than averages alone. Third, given the wide uncertainties in results from reopening, criteria should be established for rapidly detecting when to tighten precautions. Fourth, contact tracing and isolation capabilities should be ensured to be sufficient to respond to the range of model results.

Like any model-based analysis, results herein have some limitations and simplifications. A common barrier in such models is data availability for input estimation and results validation (hence our search-based approach). The deterministic ODE modeling framework ignores inherent variability and population heterogeneity [118], motivating our use of Monte Carlo analysis, parameter search replicates, and randomly sampled scenarios. Standard model simplifications include limiting the number of populations (eg, one overall homogenous community population), limiting spread to just SARS-CoV-2 (eg, ignoring seasonal influenza, substance abuse [75,76], and co-epidemic impacts), and not time-varying precaution compliance as concerns and vigilance relax or heighten over time. Some scenarios were also included for potential insights rather than being feasible in practice (eg, 75% reduction in *R_0_*, near 100% precaution compliance).

Further work could expand on these results, including addressing some of the above simplifications, rerunning analyses for future semesters using more recent data for model calibration, and considering more heterogeneity in community and student populations. Future work could also seek to determine combined conditions (reduced prevalence, vaccine effectiveness, improved precaution methods, etc) under which outcomes are both safer and more certain. Public health reopening and precaution decisions at citywide or statewide levels also might be examined, such as alternating on-campus semesters or limiting combined student densities, to manage net community risks.

### Conclusion

Controlling the COVID-19 pandemic is extremely critical. Mathematical models can offer valuable insights to inform important public health and policy decisions*,* including potential community and campus impacts from university reopening. The analysis summarized herein suggests that outcomes over a 16-week semester can be highly unpredictable under any set of assumptions or precautions, with three important implications: (1) community impacts from campus reopening are highly difficult to predict in advance, (2) on- and off-campus surveillance and response methods therefore are critical, and (3) additional precautions to reduce impacts of open campuses on local communities appear warranted.

## Appendix

Multimedia Appendix 1 General logic of campus × community two-population COVID-19 disease spread model. Susceptible: individuals not currently infected but who can become infected; exposed: individuals who are exposed and potentially infected but not yet infectious to others; infected: individuals who are infected and can infect others; recovered: individuals who were infected, survived, and cannot become reinfected nor infect others within the study time frame; dead: individuals who were infected and died from COVID-19 or complications.

Multimedia Appendix 2 Examples of COVID-19 model accuracy (2020): (A) US state (shown: Massachusetts, California, and Florida) and (B) US county (shown: Dougherty County, GA; Eagle County, CO; and Suffolk County, MA). Normalized root mean square errors, summarized in figure legends, show decent model accuracy results across multiple settings.

Multimedia Appendix 3 Impact expectation.

Multimedia Appendix 4 Impact predictability.

## Abbreviations

ODE: ordinary differential equation
