# Supplementary figures and images for: Community and Campus COVID-19 Risk Uncertainty Under University Reopening Scenarios: Model-Based Analysis
Source: JMIR Public Health Surveill. 2021 Apr 7;7(4):e24292. doi: 10.2196/24292 (PMC8030657; doi:10.2196/24292)

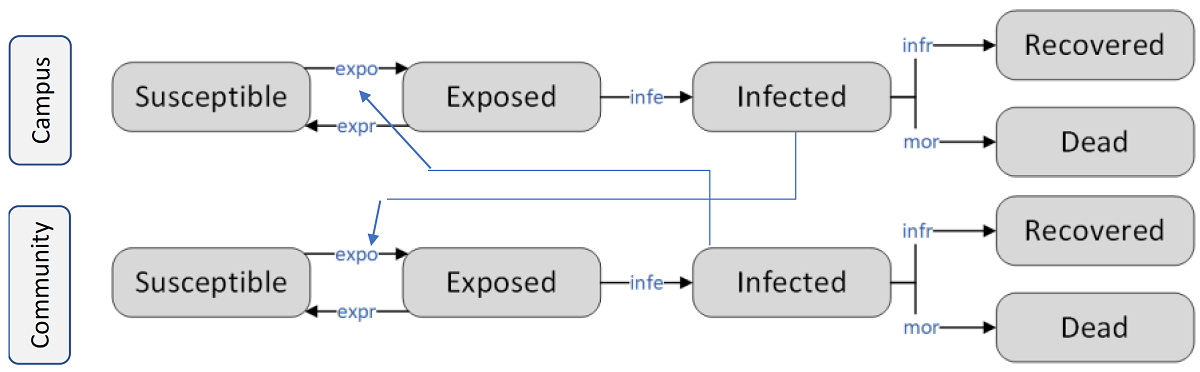

Supplement: Multimedia Appendix 1 [file publichealth_v7i4e24292_app1.png]

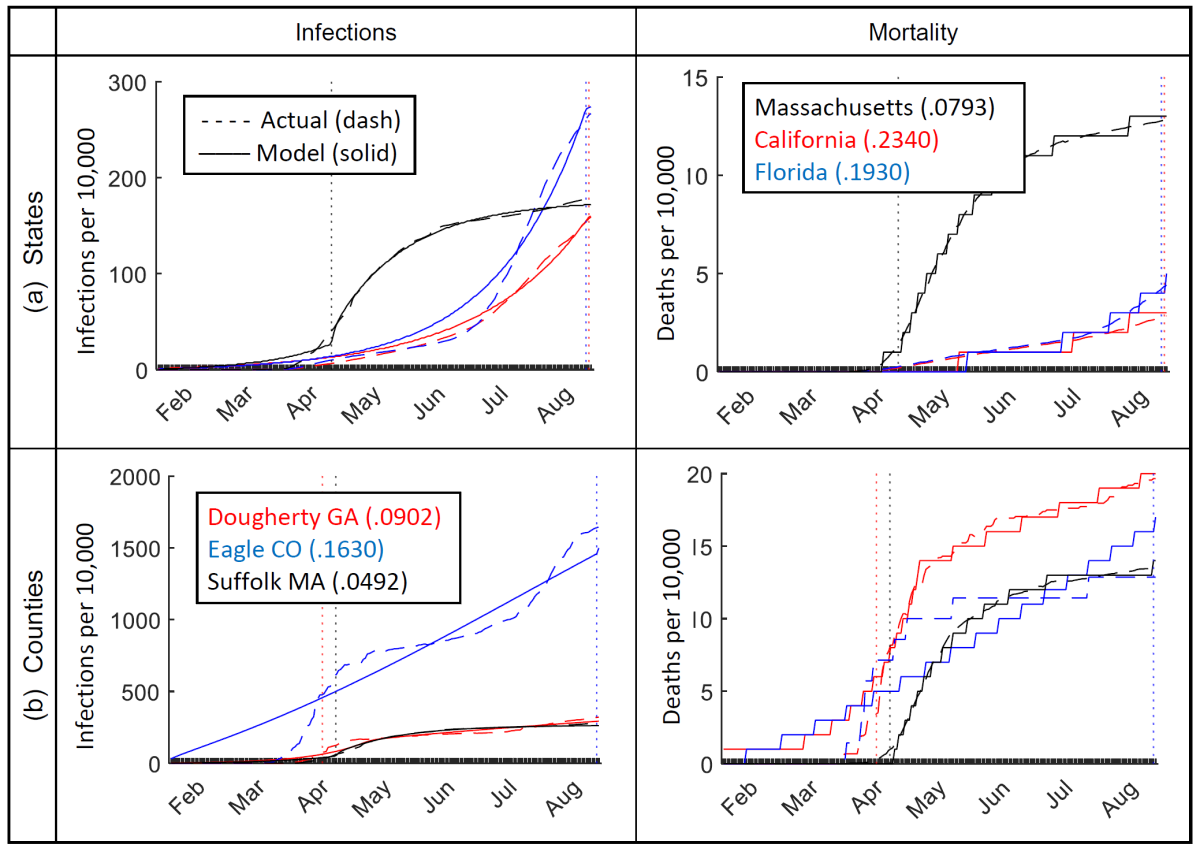

Supplement: Multimedia Appendix 2 [file publichealth_v7i4e24292_app2.png]
