# Supplementary material for: Community and Campus COVID-19 Risk Uncertainty Under University Reopening Scenarios: Model-Based Analysis
Source: JMIR Public Health Surveill. 2021 Apr 7;7(4):e24292. doi: 10.2196/24292 (PMC8030657; doi:10.2196/24292)
Supplement: Multimedia Appendix 3 [file publichealth_v7i4e24292_app3.docx]

**Multimedia Appendix 3. Impact expectation.** Sensitivity analysis of model inputs on expected values of campus and community COVID-19 total and additional infections and deaths over a 16-week semester. Tabulated values represent regression coefficient sizes normalized to their corresponding parameter ranges for main effects and pairwise interactions. Asterisks and dashes denote statistical significance (^***^, *P* < .001; ^**^, *P* < .01; ^*^, *P* < .05; °, *P* < .1; –, *P* > .1 (not significant).

|  | | **Campus** | | | | **Community** | | | |
| --- | --- | --- | --- | --- | --- | --- | --- | --- | --- |
|  | | **Infections** | | **Deaths** | | **Infections** | | **Deaths** | |
| **Factor** | | **Total**  (× 100) | **Additional**  (× 100) | **Total** | **Additional** | **Total**  (× 1,000) | **Additional**  (× 1,000) | **Total**  (× 1,000) | **Additional**  (× 100) |
| **Main effects only** | $R_{0,1}$ | 28.0^***^ | -5.78^***^ | 57.1^***^ | -6.06^***^ | 5.25^***^ | 5.35^***^ | – | 6.37^***^ |
|  | $R_{0,2}$ | 4.76^***^ | 4.76^***^ | – | 6.29^***^ | 33.0^***^ | -6.44^***^ | 5.34^***^ | -6.32^***^ |
|  | $ri$ | 1.80^***^ | 1.80^***^ | – | 2.79^**^ | 2.37^*^ | 2.37^***^ | – | 3.18^***^ |
|  | $\pi_{1}$ | 13.4^*^ | -4.11^***^ | 37.9^***^ | -4.75^***^ | 1.69° | 1.68^***^ | – | 3.04^***^ |
|  | $\pi_{2}$ | – | .789^*^ | – | 1.74° | 6.89^***^ | -3.09^***^ | 1.40^**^ | -2.97^***^ |
|  | $p$ | – | – | 78.9^***^ | 4.34^***^ | 4.50^***^ | 0.605° | 5.99^***^ | 5.01^***^ |
|  | $t_{inc}$ | 17.0^***^ | 1.55^***^ | 27.7^***^ | 3.15^**^ | 19.7^***^ | 1.22^***^ | 2.45^***^ | 3.30^***^ |
|  | $t_{rec}$ | -69.5^***^ | -5.76^***^ | -98.3^***^ | -7.02^***^ | -77.2^***^ | -6.49^***^ | -8.58^***^ | -7.53^***^ |
|  | ${CFR}_{1}$ | – | – | 110^***^ | 6.30^***^ | – | – | – | – |
|  | ${CFR}_{2}$ | – | – | – | – | 5.92^***^ | – | 8.20^***^ | 6.75^***^ |
|  | $t_{i2d}$ | – | – | -74.7^***^ | -4.27^***^ | -3.94^***^ | – | -5.44^***^ | -4.50^***^ |
| **Main effects + pairwise interactions** | $R_{0,1}$ | 91.4^***^ | -4.77^***^ | – | 9.45^*^ | 17.1^***^ | 17.1^***^ | – | – |
|  | $R_{0,2}$ | 17.9^***^ | 17.9^***^ | – | – | 97.5^**^ | -3.04^*^ | – | 12.8^**^ |
|  | $ri$ | 6.01^*^ | 6.01^***^ | – | – | 6.09^*^ | 6.09^***^ | – | – |
|  | $\pi_{1}$ | 20.9^***^ | -4.03^**^ | – | – | – | 2.61^*^ | – | – |
|  | $\pi_{2}$ | – | – | – | – | 10.4^***^ | – | – | – |
|  | $p$ | 14.4^***^ | – | 71.8^***^ | – | 11.6^***^ | – | 4.21^**^ | – |
|  | $t_{inc}$ | 46.4^***^ | 6.64^***^ | – | – | 53.5^***^ | 5.32^***^ | – | – |
|  | $t_{rec}$ | – | -5.65^***^ | 266^***^ | 12.1^**^ | 23.3^***^ | -3.70^**^ | 22.8^***^ | 16.3^***^ |
|  | ${CFR}_{1}$ | – | – | – | – | – | – | – | – |
|  | ${CFR}_{2}$ | – | – | – | – | – | – | – | – |
|  | $t_{i2d}$ | – | – | 169^***^ | – | 10.7^***^ | – | 12.0^***^ | 8.29^*^ |
|  | $R_{0,1}\times R_{0,2}$ | -9.33^***^ | -9.33^***^ | – | -10.7^***^ | -9.91^***^ | -9.91^***^ | – | -10.4^***^ |
|  | $R_{0,1}\times ri$ | -3.47^***^ | -3.47^***^ | – | -4.41^*^ | 3.22^**^ | 3.22^***^ | – | 4.84^**^ |
|  | $R_{0,1}\times\pi_{1}$ | -5.63^***^ | 8.10^***^ | – | 7.54^***^ | – | – | – | 3.53^*^ |
|  | $R_{0,1}\times\pi_{2}$ | – | -1.43^**^ | – | – | -3.96^***^ | -3.96^***^ | – | -4.31^**^ |
|  | $R_{0,1}\times p$ | -6.81^***^ | – | 76.0^***^ | -6.77^***^ | – | .913° | – | 8.41^***^ |
|  | $R_{0,1}\times t_{inc}$ | -19.3^***^ | -3.02^***^ | – | -6.79^***^ | – | – | – | 4.64^**^ |
|  | $R_{0,1}\times t_{rec}$ | -40.1^***^ | 11.6^***^ | -94.5^***^ | 12.2^***^ | -9.98^***^ | -9.98^***^ | -1.23° | -12.3^***^ |
|  | $R_{0,1}\times{CFR}_{1}$ | – | – | 101^***^ | -10.7^***^ | – | – | – | – |
|  | $R_{0,1}\times{CFR}_{2}$ | – | – | – | – | – | .887° | 1.10° | 11.0^***^ |
|  | $R_{0,1}\times t_{i2d}$ | – | – | -68.6^***^ | 7.23^***^ | – | – | – | -7.37^***^ |
|  | $R_{0,2}\times ri$ | 2.42^*^ | 2.42^***^ | – | 4.61^**^ | -4.32^***^ | -4.32^***^ | – | -4.86^**^ |
|  | $R_{0,2}\times\pi_{1}$ | -6.20^***^ | -6.20^***^ | – | -8.04^***^ | -2.72^*^ | -2.72^***^ | – | -3.54^*^ |
|  | $R_{0,2}\times\pi_{2}$ | – | – | – | – | -2.17° | 5.83^***^ | – | 3.97^**^ |
|  | $R_{0,2}\times p$ | – | – | – | 7.95^***^ | – | -1.24^**^ | 7.43^***^ | -7.69^***^ |
|  | $R_{0,2}\times t_{inc}$ | – | 1.14^*^ | – | 5.00^**^ | -24.3^***^ | -2.20^***^ | – | -7.16^***^ |
|  | $R_{0,2}\times t_{rec}$ | -9.31^***^ | -9.31^***^ | – | -12.4^***^ | -55.6^***^ | 13.1^***^ | -9.69^***^ | 12.8^***^ |
|  | $R_{0,2}\times{CFR}_{1}$ | – | – | – | 11.1^***^ | – | – | – | – |
|  | $R_{0,2}\times{CFR}_{2}$ | – | – | – | – | 10.2^***^ | -.851° | 9.32^***^ | -11.0^***^ |
|  | $R_{0,2}\times t_{i2d}$ | – | – | – | -7.54^***^ | -6.92^***^ | – | -6.35^***^ | 7.29^***^ |
|  | $ri\times\pi_{1}$ | -1.73^***^ | -1.73^***^ | – | -2.95° | – | 1.08^*^ | – | – |
|  | $ri\times\pi_{2}$ | – | – | – | – | – | -1.23^*^ | – | – |
|  | $ri\times p$ | – | – | – | 3.76^*^ | – | – | – | 4.38^**^ |
|  | $ri\times t_{inc}$ | – | – | – | – | – | – | – | – |
|  | $ri\times t_{rec}$ | -3.45^***^ | -3.45^***^ | – | -5.43^**^ | -4.35^***^ | -4.35^***^ | – | -6.05^***^ |
|  | $ri\times{CFR}_{1}$ | – | – | – | 4.92^**^ | – | – | – | – |
|  | $ri\times{CFR}_{2}$ | – | – | – | – | – | – | – | 5.52^***^ |
|  | $ri\times t_{i2d}$ | – | – | – | -3.33° | – | – | – | -3.68^*^ |
|  | $\pi_{1}\times\pi_{2}$ | – | -1.16^*^ | – | – | – | -1.53^**^ | – | – |
|  | $\pi_{1}\times p$ | – | -.889° | 45.1^***^ | -5.98^***^ | – | 1.30^**^ | – | 4.94^**^ |
|  | $\pi_{1}\times t_{inc}$ | 5.56^***^ | -5.14^***^ | – | -6.52^***^ | – | – | – | – |
|  | $\pi_{1}\times t_{rec}$ | – | 8.14^***^ | -24.8^**^ | 9.44^***^ | -2.76^*^ | -2.76^***^ | – | -5.58^***^ |
|  | $\pi_{1}\times{CFR}_{1}$ | – | – | 66.6^***^ | -8.37^***^ | – | – | – | – |
|  | $\pi_{1}\times{CFR}_{2}$ | – | – | – | – | – | – | – | 5.23^***^ |
|  | $\pi_{1}\times t_{i2d}$ | – | – | -45.0^***^ | 5.66^***^ | – | – | – | -3.42^*^ |
|  | $\pi_{2}\times p$ | – | – | – | 2.85° | – | -1.09^*^ | 1.71^**^ | -3.70^*^ |
|  | $\pi_{2}\times t_{inc}$ | – | – | – | – | 5.02^***^ | -4.70^***^ | – | -4.19^**^ |
|  | $\pi_{2}\times t_{rec}$ | – | -1.38^**^ | – | -3.28° | -2.78^*^ | 6.04^***^ | -1.39^*^ | 5.86^***^ |
|  | $\pi_{2}\times{CFR}_{1}$ | – | – | – | 3.06° | – | – | – | – |
|  | $\pi_{2}\times{CFR}_{2}$ | – | – | – | – | – | – | 2.39^***^ | -5.10^***^ |
|  | ${\pi_{2}\times t}_{i2d}$ | – | – | – | – | – | – | -1.55^*^ | 3.31^*^ |
|  | $p\times t_{inc}$ | -3.44^***^ | – | 33.2^***^ | 4.67^**^ | – | 1.14^*^ | 3.03^***^ | 5.13^***^ |
|  | $p\times t_{rec}$ | -10.5^***^ | – | -159^***^ | -8.83^***^ | -16.8^***^ | -1.65^***^ | -12.5^***^ | -10.3^***^ |
|  | $p\times{CFR}_{1}$ | – | – | 139^***^ | 7.65^***^ | – | – | – | – |
|  | $p\times{CFR}_{2}$ | – | – | – | – | 7.22^***^ | .831° | 10.3^***^ | 8.67^***^ |
|  | $p\times t_{i2d}$ | – | – | -94.0^***^ | -5.16^**^ | -4.71^***^ | – | -6.72^***^ | -5.74^***^ |
|  | $t_{inc}\times t_{rec}$ | -20.1^***^ | -2.88^***^ | -40.4^***^ | -6.11^***^ | -29.7^***^ | -1.89^***^ | -4.01^***^ | -6.17^***^ |
|  | $t_{inc}\times{CFR}_{1}$ | – | – | 49.0^***^ | 5.57^**^ | – | – | – | – |
|  | $t_{inc}\times{CFR}_{2}$ | – | – | – | – | 4.08^***^ | .918° | 4.27^***^ | 5.78^***^ |
|  | $t_{inc}\times t_{i2d}$ | – | – | -33.3^***^ | -3.80^*^ | -2.73^*^ | – | -2.89^***^ | -3.98^**^ |
|  | $t_{rec}\times{CFR}_{1}$ | – | – | -173^***^ | -12.4^***^ | – | – | – | – |
|  | $t_{rec}\times{CFR}_{2}$ | – | – | – | – | -10.6^***^ | -1.09^*^ | -14.8^***^ | -13.0^***^ |
|  | $t_{rec}\times t_{i2d}$ | – | – | 117^***^ | 8.39^***^ | 7.05^***^ | – | 9.84^***^ | 8.70^***^ |
|  | ${CFR}_{1}\times{CFR}_{2}$ | – | – | – | – | – | – | – | – |
|  | ${CFR}_{1}\times t_{i2d}$ | – | – | -132^***^ | -7.53^***^ | – | – | – | – |
|  | ${CFR}_{2}\times t_{i2d}$ | – | – | – | – | -6.76^***^ | – | -9.33^***^ | -7.75^***^ |
