# Supplementary material for: Community and Campus COVID-19 Risk Uncertainty Under University Reopening Scenarios: Model-Based Analysis
Source: JMIR Public Health Surveill. 2021 Apr 7;7(4):e24292. doi: 10.2196/24292 (PMC8030657; doi:10.2196/24292)
Supplement: Multimedia Appendix 4 [file publichealth_v7i4e24292_app4.docx]

**Multimedia Appendix 4. Impact predictability.** Sensitivity analysis of model inputs on variability of campus and community COVID-19 total and additional infections and deaths over a 16-week semester. Tabulated values represent regression coefficient sizes normalized to their corresponding parameter ranges for main effects and pairwise interactions. Asterisks and dashes denote statistical significance (^***^, *P* < .001; ^**^, *P* < .01; ^*^, *P* < .05; °, *P* < .1; –, *P* > .1 (not significant).

|  | | **Campus** | | | | **Community** | | | |
| --- | --- | --- | --- | --- | --- | --- | --- | --- | --- |
|  | | **Infections** | | **Deaths** | | **Infections** | | **Deaths** | |
| **Factor** | | **Total**  (× 100) | **Additional**  (× 100) | **Total** | **Additional** | **Total**  (× 1,000) | **Additional**  (× 1,000) | **Total**  (× 1,000) | **Additional**  (× 100) |
| **Main effects only** | $R_{0,1}$ | -3.81^***^ | -3.08^***^ | 28.8^***^ | -4.26^***^ | -2.10^***^ | 2.77^***^ | – | 4.23^***^ |
|  | $R_{0,2}$ | -1.99^***^ | 3.02^***^ | – | 4.39^***^ | -4.67^***^ | -2.94^***^ | 2.67^***^ | -4.23^***^ |
|  | $ri$ | -.99^**^ | 1.45^***^ | – | 2.31^**^ | -.995° | 1.93^***^ | – | 2.56^**^ |
|  | $\pi_{1}$ | -5.88^***^ | -2.97^***^ | 8.90^*^ | -3.87^***^ | – | .681^**^ | – | 1.88^*^ |
|  | $\pi_{2}$ | – | – | – | – | -2.27^***^ | -2.33^***^ | – | -2.34^**^ |
|  | $p$ | 1.61^***^ | .460° | 46.5^***^ | 3.89^***^ | 6.51^***^ | .817^***^ | 4.08^***^ | 4.37^***^ |
|  | $t_{inc}$ | -6.77^***^ | 1.75^***^ | 13.0^**^ | 3.19^***^ | -9.41^***^ | 1.04^***^ | .928^**^ | 3.09^***^ |
|  | $t_{rec}$ | -5.04^***^ | -3.09^***^ | -38.1^***^ | -4.35^***^ | -6.2^***^ | -2.92^***^ | -2.95^***^ | -4.41^***^ |
|  | ${CFR}_{1}$ | – | – | 37.7^***^ | 4.66^***^ | – | – | – | – |
|  | ${CFR}_{2}$ | – | – | – | – | 4.35^***^ | – | 3.11^***^ | 4.79^***^ |
|  | $t_{i2d}$ | – | – | -46.7^***^ | -3.85^***^ | -3.21^***^ | – | -3.58^***^ | -3.84^***^ |
| **Main effects + pairwise interactions** | $R_{0,1}$ | -29.6^***^ | – | -38.0^**^ | 12.6^**^ | -15.2^***^ | 6.35^***^ | – | – |
|  | $R_{0,2}$ | -14.0^***^ | 8.03^***^ | – | – | -47.0^***^ | 2.59^**^ | -4.69^***^ | 16.1^***^ |
|  | $ri$ | -5.58^***^ | 5.22^***^ | – | – | -6.25^***^ | 5.84^***^ | – | – |
|  | $\pi_{1}$ | -15.3^***^ | -1.42^*^ | – | 7.82° | -3.70^*^ | – | – | – |
|  | $\pi_{2}$ | – | – | – | – | -8.54^***^ | – | – | – |
|  | $p$ | -3.68^**^ | – | 48.1^***^ | – | -5.18^***^ | 1.39^*^ | 3.46^***^ | – |
|  | $t_{inc}$ | -35.4^***^ | 4.23^***^ | – | – | -49.5^***^ | 2.77^***^ | -3.27^**^ | – |
|  | $t_{rec}$ | -27.3^***^ | – | 141^***^ | 12.1^**^ | -15.8^***^ | 1.86^*^ | 13.0^***^ | 15.6^***^ |
|  | ${CFR}_{1}$ | – | – | -55.9^***^ | – | – | – | – | – |
|  | ${CFR}_{2}$ | – | – | – | – | -3.97^*^ | – | -6.32^***^ | – |
|  | $t_{i2d}$ | – | – | 211^***^ | 8.75^*^ | 11.9^***^ | – | 17.4^***^ | 11.3^***^ |
|  | $R_{0,1}\times R_{0,2}$ | 4.85^***^ | -6.14^***^ | – | -8.61^***^ | 5.85^***^ | -5.62^***^ | – | -8.09^***^ |
|  | $R_{0,1}\times ri$ | 2.30^***^ | -2.98^***^ | – | -4.42^**^ | – | 3.14^***^ | – | 4.82^***^ |
|  | $R_{0,1}\times\pi_{1}$ | 2.28^***^ | 6.08^***^ | 20.8^***^ | 7.87^***^ | – | 1.65^***^ | – | 4.15^**^ |
|  | $R_{0,1}\times\pi_{2}$ | 1.16^*^ | -.782^**^ | – | – | 1.26° | -4.14^***^ | – | -4.46^***^ |
|  | $R_{0,1}\times p$ | 1.90^***^ | -.888^**^ | 72.2^***^ | -7.93^***^ | – | 1.32^***^ | – | 8.23^***^ |
|  | $R_{0,1}\times t_{inc}$ | 12.6^***^ | -3.50^***^ | 12.8^*^ | -6.26^***^ | 5.99^***^ | 2.13^***^ | – | 5.97^***^ |
|  | $R_{0,1}\times t_{rec}$ | 10.8^***^ | 6.11^***^ | -60.6^***^ | 8.45^***^ | 4.34^***^ | -5.60^***^ | – | -8.73^***^ |
|  | $R_{0,1}\times{CFR}_{1}$ | – | – | 64.1^***^ | -9.22^***^ | – | – | – | – |
|  | $R_{0,1}\times{CFR}_{2}$ | – | – | – | – | – | .558° | – | 9.35^***^ |
|  | $R_{0,1}\times t_{i2d}$ | – | – | -73.9^***^ | 7.39^***^ | – | – | – | -7.21^***^ |
|  | $R_{0,2}\times ri$ | – | 2.73^***^ | – | 4.58^**^ | 2.66^***^ | -3.91^***^ | – | -4.89^***^ |
|  | $R_{0,2}\times\pi_{1}$ | 1.97^***^ | -5.83^***^ | – | -8.01^***^ | 1.89^**^ | -1.51^***^ | – | -4.06^**^ |
|  | $R_{0,2}\times\pi_{2}$ | – | .812^**^ | – | 2.66° | 1.35^*^ | 4.87^***^ | 1.06^*^ | 4.51^***^ |
|  | $R_{0,2}\times p$ | 1.30^*^ | .779^**^ | – | 7.65^***^ | 10.7^***^ | -1.94^***^ | 6.91^***^ | -8.64^***^ |
|  | $R_{0,2}\times t_{inc}$ | 4.28^***^ | 4.10^***^ | – | 6.64^***^ | 20.0^***^ | -1.88^***^ | 2.35^***^ | -6.15^***^ |
|  | $R_{0,2}\times t_{rec}$ | 4.32^***^ | -6.15^***^ | – | -8.95^***^ | 7.37^***^ | 5.77^***^ | -5.92^***^ | 8.25^***^ |
|  | $R_{0,2}\times{CFR}_{1}$ | – | – | – | 9.51^***^ | – | – | – | – |
|  | $R_{0,2}\times{CFR}_{2}$ | – | – | – | – | 8.80^***^ | -.930^**^ | 6.04^***^ | -9.37^***^ |
|  | $R_{0,2}\times t_{i2d}$ | – | – | – | -7.58^***^ | -6.46^***^ | .675^*^ | -6.40^***^ | 7.21^***^ |
|  | $ri\times\pi_{1}$ | .88° | -2.71^***^ | – | -3.36^*^ | – | 1.11^***^ | – | – |
|  | $ri\times\pi_{2}$ | – | .515° | – | – | – | -2.39^***^ | – | – |
|  | $ri\times p$ | – | – | – | 3.86^*^ | – | – | – | 4.60^***^ |
|  | $ri\times t_{inc}$ | 2.21^***^ | -.756^**^ | – | – | 3.34^***^ | -.735^*^ | – | 2.29° |
|  | $ri\times t_{rec}$ | 2.05^***^ | -3.00^***^ | – | -4.75^**^ | 1.94^**^ | -3.90^***^ | – | -5.32^***^ |
|  | $ri\times{CFR}_{1}$ | – | – | – | 4.83^**^ | – | – | – | – |
|  | $ri\times{CFR}_{2}$ | – | – | – | – | – | – | – | 5.49^***^ |
|  | $ri\times t_{i2d}$ | – | – | – | -4.06^**^ | – | – | – | -4.05^**^ |
|  | $\pi_{1}\times\pi_{2}$ | – | -.626^*^ | – | – | – | -.975^**^ | – | – |
|  | $\pi_{1}\times p$ | – | -.679^*^ | 23.0^***^ | -6.41^***^ | – | .905^**^ | – | 3.72^**^ |
|  | $\pi_{1}\times t_{inc}$ | 3.07^***^ | -3.15^***^ | – | -5.79^***^ | – | – | – | – |
|  | $\pi_{1}\times t_{rec}$ | 5.13^***^ | 6.03^***^ | -48.7^***^ | 7.77^***^ | 1.34^*^ | -1.48^***^ | – | -4.11^**^ |
|  | $\pi_{1}\times{CFR}_{1}$ | – | – | 19.0^***^ | -8.25^***^ | – | – | – | – |
|  | $\pi_{1}\times{CFR}_{2}$ | – | – | – | – | – | – | – | 4.16^**^ |
|  | $\pi_{1}\times t_{i2d}$ | – | – | -23.4^***^ | 6.18^***^ | – | – | – | -2.99^*^ |
|  | $\pi_{2}\times p$ | – | – | – | – | 3.47^***^ | -1.28^***^ | 1.38^**^ | -3.96^**^ |
|  | $\pi_{2}\times t_{inc}$ | – | – | – | – | – | -3.01^***^ | – | -3.46^**^ |
|  | $\pi_{2}\times t_{rec}$ | 1.04^*^ | -.828^**^ | – | – | -1.50^*^ | 4.69^***^ | -2.05^***^ | 4.71^***^ |
|  | $\pi_{2}\times{CFR}_{1}$ | – | – | – | 2.52° | – | – | – | – |
|  | $\pi_{2}\times{CFR}_{2}$ | – | – | – | – | – | -.722^*^ | 1.05^*^ | -5.09^***^ |
|  | ${\pi_{2}\times t}_{i2d}$ | – | – | – | – | – | – | -.921^*^ | 3.66^**^ |
|  | $p\times t_{inc}$ | 4.4^***^ | 1.19^***^ | 31.5^***^ | 5.22^***^ | 7.07^***^ | 1.33^***^ | 2.53^***^ | 5.69^***^ |
|  | $p\times t_{rec}$ | – | -.785^**^ | -84.0^***^ | -7.62^***^ | -5.71^***^ | -1.55^***^ | -7.26^***^ | -8.41^***^ |
|  | $p\times{CFR}_{1}$ | – | – | 104^***^ | 8.23^***^ | – | – | – | – |
|  | $p\times{CFR}_{2}$ | – | – | – | – | 8.66^***^ | – | 9.15^***^ | 9.42^***^ |
|  | $p\times t_{i2d}$ | – | – | -116^***^ | -5.78^***^ | -6.33^***^ | – | -9.59^***^ | -6.67^***^ |
|  | $t_{inc}\times t_{rec}$ | 9.19^***^ | -3.64^***^ | -27.8^***^ | -6.56^***^ | 11.7^***^ | -2.20^***^ | -2.44^***^ | -6.48^***^ |
|  | $t_{inc}\times{CFR}_{1}$ | – | – | 28.7^***^ | 6.63^***^ | – | – | – | – |
|  | $t_{inc}\times{CFR}_{2}$ | – | – | – | – | – | .660^*^ | 2.10^***^ | 6.51^***^ |
|  | $t_{inc}\times t_{i2d}$ | – | – | -33.0^***^ | -4.52^**^ | – | -.509° | -1.83^***^ | -4.48^***^ |
|  | $t_{rec}\times{CFR}_{1}$ | – | – | -86.6^***^ | -9.50^***^ | – | – | – | – |
|  | $t_{rec}\times{CFR}_{2}$ | – | – | – | – | -8.91^***^ | -.702^*^ | -6.85^***^ | -9.84^***^ |
|  | $t_{rec}\times t_{i2d}$ | – | – | 104^***^ | 7.84^***^ | 6.52^***^ | .522° | 7.74^***^ | 7.87^***^ |
|  | ${CFR}_{1}\times{CFR}_{2}$ | – | – | – | – | – | – | – | – |
|  | ${CFR}_{1}\times t_{i2d}$ | – | – | -102^***^ | -8.15^***^ | – | – | – | – |
|  | ${CFR}_{2}\times t_{i2d}$ | – | – | – | – | -6.68^***^ | – | -8.03^***^ | -8.28^***^ |
